# Nitric Oxide Prevents Fe Deficiency-Induced Photosynthetic Disturbance, and Oxidative Stress in Alfalfa by Regulating Fe Acquisition and Antioxidant Defense

**DOI:** 10.3390/antiox10101556

**Published:** 2021-09-29

**Authors:** Md Atikur Rahman, Ahmad Humayan Kabir, Yowook Song, Sang-Hoon Lee, Mirza Hasanuzzaman, Ki-Won Lee

**Affiliations:** 1 Grassland and Forage Division, National Institute of Animal Science, Rural Development Administration, Cheonan 31000, Korea; atikbt@korea.kr (M.A.R.); songs0806@korea.kr (Y.S.); sanghoon@korea.kr (S.-H.L.); 2 Molecular Plant Physiology Laboratory, Department of Botany, University of Rajshahi, Rajshahi 6205, Bangladesh; ahmad.kabir@ru.ac.bd; 3 Department of Genetics, University of Georgia, Athens, GA 30602, USA; 4 Department of Agronomy, Faculty of Agriculture, Sher-e-Bangla Agricultural University, Sher-e-Bangla Nagar, Dhaka 1207, Bangladesh; mhzsauag@yahoo.com

**Keywords:** nitric oxide, iron-deficiency, chlorosis, antioxidant, alfalfa

## Abstract

Iron (Fe) deficiency impairs photosynthetic efficiency, plant growth and biomass yield. This study aimed to reveal the role of nitric oxide (NO) in restoring Fe-homeostasis and oxidative status in Fe-deficient alfalfa. In alfalfa, a shortage of Fe negatively affected the efficiency of root andshoot length, leaf greenness, maximum quantum yield PSII (Fv/Fm), Fe, S, and Zn accumulation, as well as an increase in H_2_O_2_ accumulation. In contrast, in the presence of sodium nitroprusside (SNP), a NO donor, these negative effects of Fe deficiency were largely reversed. In response to the SNP, the expression of Fe transporters (*IRT1*, *NRAMP1*) and S transporter (*SULTR1;2*) genes increased in alfalfa. Additionally, the detection of NO generation using fluorescence microscope revealed that SNP treatment increased the level of NO signal, indicating that NO may act as regulatory signal in response to SNP in plants. Interestingly, the increase of antioxidant genes and their related enzymes (Fe-SOD, APX) in response to SNP treatment suggests that Fe-SOD and APX are key contributors to reducing ROS (H_2_O_2_) accumulation and oxidative stress in alfalfa. Furthermore, the elevation of Ascorbate-glutathione (AsA-GSH) pathway-related genes (*GR* and *MDAR*) Fe-deficiency with SNP implies that the presence of NO relates to enhanced antioxidant defense against Fe-deficiency stress.

## 1. Introduction

Iron (Fe) is an essential micro-nutrient for plants, as it participates in numerous physiological processes such as photosynthesis, respiration, and nitrogen assimilation [1]. Therefore, any restriction in Fe acquisition hampers plant growth, development, and productivity [2]. In calcareous soils or at high pH levels, Fe is readily oxidized which forms insoluble ferric oxide (Fe^3+^), resulting in Fe (Fe^2+^) deficiency-induced growth inhibition and leaf chlorosis [1]. To deal with this problem, the plant evolved two strategies to deal with soluble Fe (Fe^2+^) shortage and recover Fe from soils. First, there are strategy-I plants (all dicots and non-graminaceous monocots), in which Fe^3+^ is reduced into Fe^2+^ by a plasma membrane ferric reductase enzyme encoded by the *FRO* (ferric reduction oxidase) gene, before being transported across the rhizodermis cell by a Fe^2+^ transporter, encoded by an *IRT* (iron-regulated transporter) gene [1,3].

On the other hand, strategy-II plants produce phytosiderophores (PS) capable of chelating Fe^3+,^ which are then taken up by specific epidermal root cell plasma membrane transporters [4]. However, alfalfa is strategy-I species. Under Fe-deficient conditions, strategy-I species undergo several morphological and physiological changes to aid in nutrient transportation and acquisition. For example, high expression of *IRT1* gene enhances Fe^2+^ acquisition in plants [3]. Similarly, the up-regulation of Fe responsive genes *IRT1* and *FRO1* in *Brassica* at an early stage of S deficiency has been reported [5]. Furthermore, Fe deficiency in plants can cause oxidative stress at the cellular level by impairing photosystem II efficiency, increasing H_2_O_2_ levels, cellular injury, and disrupting redox homeostasis, all of which can lead to programmed cell death (PCD) [6]. In contrast, Fe deficiency was found to regulate antioxidant mechanisms in *Prunus* rootstocks, where superoxide dismutase (SOD), peroxidase (POD), and catalase (CAT) activities were differentially induced [7].

Nitric oxide (NO) is a small gaseous signaling molecule, involved in the alleviation of oxidative stress, induction of antioxidant activity, and plant sustenance against environmental stimuli [8]. An increasing number of studies have revealed the protective role of NO in plant tolerance to different abiotic stresses. In response to salt stress, NO supplementation regulates SOD, CAT, and osmolyte activities in chickpea [9]. It was also reported that NO donor sodium nitroprusside (SNP) could act as an antioxidant in barley, leading to prevention of PCD [10]. In response to cadmium stress, exogenous NO increases chlorophyll and the Chl a/b ratio in *Brassica* [11]. Despite these significant advances in NO response in multiple stress tolerance in plants, little is known about NO response in nutrient-deficient/nutrient sufficient conditions. Therefore, it is imperative to investigate NO-mediated Fe-homeostasis, alleviation of Fe-induced oxidative stress, and balancing of redox state in plants under Fe-deficiency.

Alfalfa (*Medicago sativa* L.) is a perennial legume crop that serves as nutritious fodder for livestock. Nutrient deficiency negatively impacts on forage growth, biomass yield, quality parameters, digestibility, and ultimate animal performance. It is therefore critical to investigate long-term strategies for maintaining nutrient content in forage in changing climates. Alfalfa is also a good source of protein; it is well known that Fe acts as a cofactor in various enzymes and proteins [12]. Unfortunately, Fe deficiency causes chlorosis, a decrease in protein and chlorophyll content, and reduces in forage yield and quality, especially in alkaline soils [13]. Therefore, considering the above facts the aim of the study was designed to explore NO-mediated mechanisms associated with plant protection from Fe-induced chlorosis, photosynthetic disruption, transcriptional regulation of Fe-responsive genes, and antioxidant defense.

## 2. Materials and Methods

### 2.1. Plant Cultivation and Treatment

Viable seeds of alfalfa (*Medicago sativa* L.) were treated with 70% ethanol for 1 min, washed properly using deionized water, and then placed for germination up to 2–3 days. Five-day-old seedlings were transferred into plastic boxes supplemented with micro and macro-elements [14]. There were four treatments: control (25 µM FeNaEDTA); −Fe (0.1 µM FeNaEDTA); −Fe (0.1 µM FeNaEDTA) and sodium nitroprusside (SNP; 100 µM) as nitric oxide (NO) donor; and SNP (100 µM). The plants were maintained at 25 °C under 60–65% relative humidity, 200 μmolm^−2^ s^−1^ light intensity and light/dark cycle (14 h/10 h dark). The plants were harvested 2 weeks following treatments.

### 2.2. Measurement of Morphological Features and Photosynthetic Parameters

The root and shoot lengths were measured in centimeters (cm) scale. The fresh weight (FW) of the plants was determined using a digital balance. Alfalfa plants were kept at dark for 1 h before physiological indices were measured. The leaf greenness of young alfalfa leaves was determined using a SPAD meter (Minolta, Japan). The maximum yield of photosystem II (PSII; Fv/Fm) was determined using a portable fluorometer after plants were dark-adapted for 20 min at room temperature (PAM 200, Effeltrich, Germany).

### 2.3. Nitric oxide (NO) Localization Using Fluorescent Histochemical Staining

Endogenous NO formation in root tips was measured using 4,5-diaminofluore scein diacetate (DAF-2DA) (Sigma-Aldrich, Burlington, MA, USA) as a NO-specific fluorescent dye [15]. In brief, dimethyl sulfoxide (DMSO) was used to prepare 10 μM DAF-2DA. Root tips of alfalfa were soaked in 10 mM Tris−HCl buffer (pH 6.5) containing 10 μM DAF-2DA for 30 min at dark conditions. The incubated root tips were washed with diethyl pyrocarbonate (DEPC) treated water and observed using a fluorescence microscope (Logos Biosystems, Anyang, South Korea) with 495 nm excitation and 515 nm emissions.

### 2.4. Estimation of Elemental Concentration

Following treatments, alfalfa roots were washed with deionized water to remove nutrient components from the surface area, and excess water was blotted with tissue paper. Root and shoot were dried for 72 h at 70 °C. Equivalent amounts of plant samples were weighed and digested with a solution (HClO_4_/HNO_3_; 1:3 *v*/*v*), and elements were measured using inductively coupled plasma mass spectroscopy (ICP-MS, Agilent 7700, Santa Clara, CA, USA). To prepare with a standard curve, a multi-element ICP-standard-solution (ROTI^®^STAR, Roth, Germany) was considered Elements were analyzed from samples of the three biological replications.

### 2.5. Analysis of Soluble Protein Content

The amount of soluble protein was determined according to the protocol described previously [16]. Shortly, 100 mg of plant tissue was homogenized with Tris-HCl (50 mM, pH 7.5), EDTA (2 mM), and 0.04% (*v*/*v*) β-mercaptoethanol (β-ME). The mixture was centrifuged at 10,000 rpm for 15 min. Following that, 1 mL supernatant was mixed with 1 mL Coomassie Brilliant Blue (CBB), and the absorbance was measured at 595 nm.

### 2.6. Hydrogen Peroxide Accumulation

The accumulation of hydrogen peroxide (H_2_O_2_) was detected spectrophotometrically using the previously described protocol [17]. Shortly, 100 mg of ground sample was homogenized with KP-buffer (50 mM, pH adjusted to 7.0) containing catalase inhibitor hydroxylamine (1 mM). The mixture was centrifuged for 20 min at 12,000 rpm then supernatant (0.7 mL) was transferred to a new tube and 0.7 mL 20% H_2_SO_4_ containing titanium chloride (TiCl) was added. The mixture was centrifuged at 12,000 rpm for 15 min. Finally, 1 mL of supernatant was taken and measured with absorbance at 410nm using a spectrophotometer (UV-1650PC, Shimadzu, Japan).

### 2.7. Measurement of Cell Death

Cell death percentages (%) were measured according to the method used earlier [18]. Shortly, 200 mg of plant tissue was homogenized with Evan’s blue solution (2 mL) for 15 min. The mixture was treated with 80% ethanol for 8–10 min. The solution was incubated at 50 °C for 20 min in a water bath (Vision Scientific, Daejeon, Korea) system. The mixture was centrifuged at 12,000 rpm for 10 min. The supernatant (1 mL) was then exposed to a wavelength of at 600 nm. The percentage of cell death of tissue was calculated according to the fresh weight basis.

### 2.8. Gene Expression Analysis by Real-Time PCR

The RNeasy plant mini kit was used to isolate total RNA from plant tissue (QIAGEN, Hilden, Germany). Shortly, 0.1 g of ground tissue was mixed with RNA extraction buffer containing 2M DDT and 1% (*v*/*v*) β-ME. The mixture was vortex thoroughly before being centrifuged at 13,000 rpm for 2 min. Following multiple centrifugation and washing steps, total RNA yield was obtained. The RNA concentration in the sample was determined using a nano-drop UV/Vis spectrophotometer (UVISDrop-99, Taipei, Taiwan). For further molecular analyses, RNA concentrations of more than 300 ng/μL were considered. The cDNA synthesis kit (Bio-Rad, USA) was used for the synthesis of cDNA. The CFX96 Real-Time system (Bio-Rad, USA) was used to analyze gene expression. The gene-specific primers were used for qPCR (Supplementary Table S1) analysis. The reaction mixture (20 μL) contained 10 μL of SYBR Green, 1μL of cDNA, 1μL of each forward and reverse primer (10 μM), and rest of DEPC treated water. The qPCR system was set to 95 °C for 3 min, 40 amplification cycles of 5 s at 95 °C, 30 sec of annealing 60 °C, and 5 min of extension at 60 °C. The expression of the target genes was analyzed using the dd^−∆Ct^ method [19], where *actin* was considered as an internal control.

### 2.9. Antioxidant Enzyme Activity

Antioxidant enzyme activities of plant tissue were measured following the protocol used previously [20]. Briefly, 100 mg tissue was homogenized in 0.5 mL of 100 mM (KP-buffer, pH 7.0), vortex well. Then the solution was centrifuged at 10,000 rpm for 15 min, and this supernatant was used for further enzymatic analysis. In order to assess SOD, 100 µL extract was added to EDTA (0.1 mM), NaHCO_3_ (50 mM, pH 9.8) and epinephrine (0.6 mM). The adrenochrome was confirmed by exposing the solution at 475 nm. The activity of APX was determined according to the method used previously [21]. The reaction buffer consisted of100 µL of sample extract, EDTA (0.1 mM), KP-buffer (50 mM, pH 7.0), hydrogen peroxide (0.1 mM), and ascorbic acid (0.5 mM). The 1 mL supernatant was taken and the absorbance was measured at 290 nm and the activity was calculated at extinction co-efficient (2.8 mM^−1^ cm^−1^). The CAT activity was measured using a mixture containing KP-buffer (100 mM, pH 7.0), hydrogen peroxide (6%), and 100 µL sample extract, and the mixture was read at 240 nm (extinction co-efficient 0.036 mM^−1^ cm^−1^) considering between 30s-60s. For GR activity, 100 μL plant extract was added to KP-buffer (100 mM), EDTA (1 mM), GSSG (20 mM) and NADPH (0.2 mM). The reaction was triggered with GSSG, which was reduced in absorbance at 340 nm in response to NADPH oxidation. The GR accumulation was ascertained using the extinction co-efficient of 6.12 mM^−1^ cm^−1^ [22].

### 2.10. Statistical Analysis

All experiments were conducted with the three biological replications for each sampling. The significance level (*p* ≤ 0.05) was considered by one-way analysis of variance (ANOVA) followed by Tukey honestly significant test, which was followed by the software SPSS Statistics 20.0. The software GraphPad Prism (version 6.0) was used for graphical presentation.

## 3. Results

### 3.1. Alteration of Morphological Features

**Figure 1:**
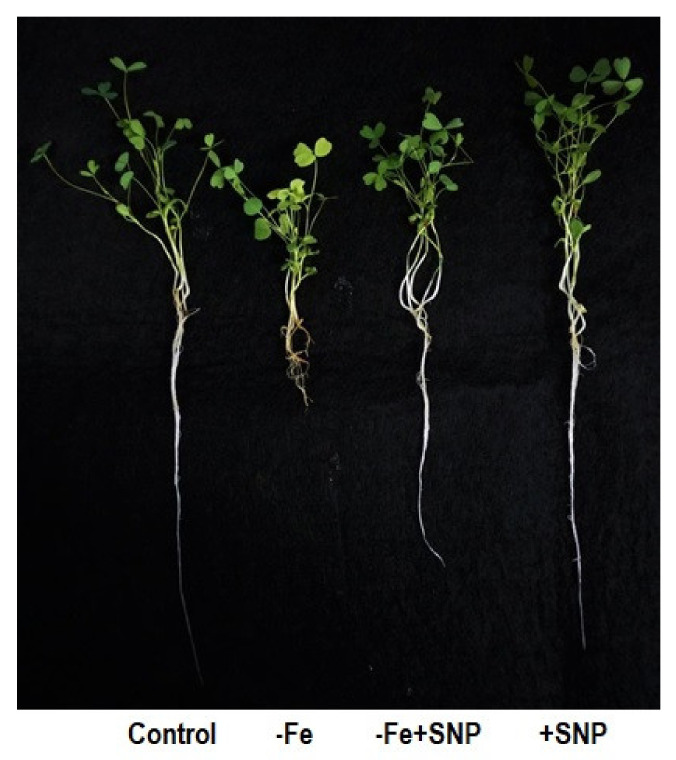
Morphological changes in alfalfa cultivated with different growth conditions: control (25 µM FeNaEDTA); −Fe (0.1 µM FeNaEDTA); −Fe (0.1 µM FeNaEDTA) and sodium nitroprusside (SNP, 100 µM) as nitric oxide (NO) donor; and SNP (100 µM).

**Figure 2:**
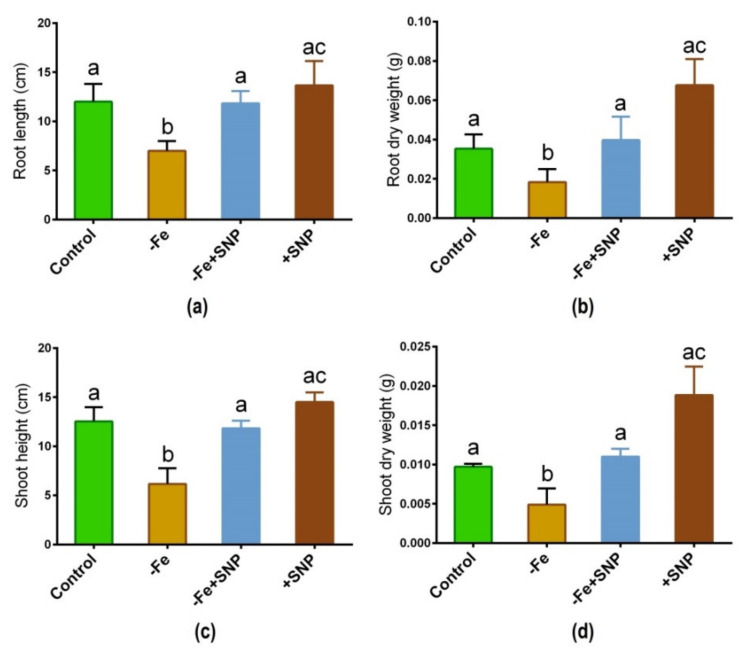
Root length (**a**), root dry weight (**b**), shoot height (**c**), and shoot dry weight (**d**) in alfalfa cultivated with different growth conditions: control (25 µM FeNaEDTA); −Fe (0.1 µM FeNaEDTA); −Fe (0.1 µM FeNaEDTA) and sodium nitroprusside (SNP, 100 µM) as nitric oxide (NO) donor; and SNP (100 µM). Different letters above the error bar indicate significant differences (*p* < 0.05) among means ± SD of treatments (*n* = 3).

Iron deficiency-significantly altered the morphological features in alfalfa following 14 days of plant cultivation in the medium. Fe deficiency inhibited the growth of alfalfa seedlings compared with those growth was sufficiently stimulated with SNP (Figure 1). Root length, root dry weight, shoot length, and shoot dry weight of SNP-supplied plants (−Fe+SNP) were significantly higher than those of Fe-deficient plants (Figure 2a–d). However, the addition of SNP to the control plants (+SNP) resulted in an increase in biomass production.

### 3.2. Fe Deficiency-Induced Chlorosis and Regulation of Photosynthetic Parameters

**Figure 3:**
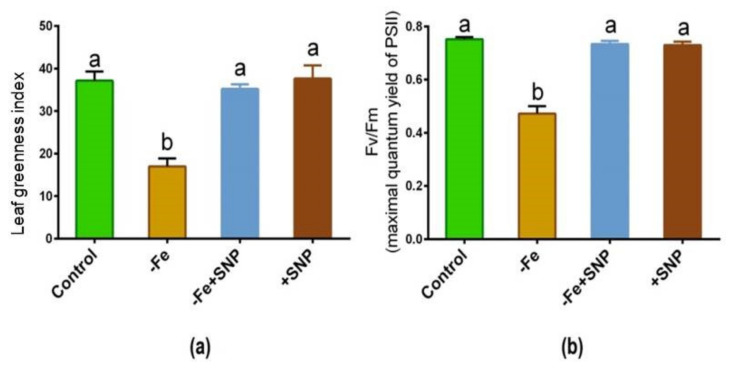
Leaf greenness (**a**) and maximum quantum yield of PSII (**b**) in alfalfa cultivated with different growth conditions: control (25 µM FeNaEDTA); −Fe (0.1 µM FeNaEDTA); −Fe (0.1 µM FeNaEDTA) and sodium nitroprusside (SNP, 100 µM) as nitric oxide (NO) donor; and SNP (100 µM). Different letters above the error bar indicate significant differences (*p* < 0.05) among means ± SD of treatments (*n* = 3).

Fe-deficient alfalfa plants exhibited chlorosis and photosynthetic disturbance, and reduced chlorophyll levels. As shown in Figure 3a,b, the leaf greenness and maximum quantum yield of PSII were significantly reduced in response to Fe deficiency, whereas these parameters were improved following exogenous SNP supplementation to the Fe deficient condition (−Fe+SNP). These parameters remained unchanged while the control plants were treated with SNP (+SNP) and untreated control.

### 3.3. Changes of Cellular Stress Indicators

**Figure 4:**
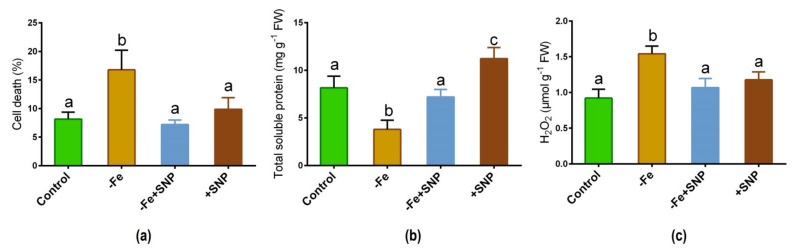
Cell death (**a**), total soluble protein (**b**), and hydrogen peroxide (H_2_O_2_) (**c**) in alfalfa cultivated with different growth conditions: control (25 µM FeNaEDTA); −Fe (0.1 µM FeNaEDTA); −Fe (0.1 µM FeNaEDTA) and sodium nitroprusside (SNP, 100 µM) as nitric oxide (NO) donor; and SNP (100 µM). Different letters above the error bar indicate significant differences (*p* < 0.05) among means ± SD of treatments (*n* = 3).

Fe deficiency caused a significant increase in cell death (%) compared to the plants treated with SNP along with non-treated control (Figure 4a). However, cell death (%) was found to be reduced after adding SNP with Fe-deficiency. Total soluble protein was significantly reduced in Fe deficient plants while it was increased in SNP-treated plants (Figure 4b). Furthermore, Fe deficiency increased the rate of H_2_O_2_ production in alfalfa while it showed a significant reduction in SNP-treated with Fe-deficiency plants (Figure 4c).

### 3.4. Regulation of Endogenous NO Level

**Figure 5:**
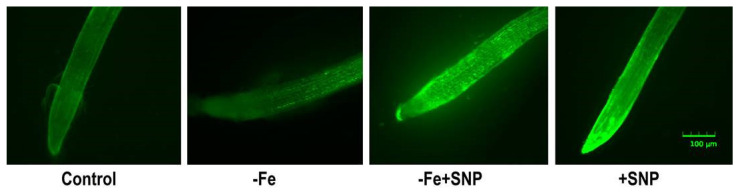
Endogenous accumulation of nitric oxide (NO) in alfalfa cultivated with different growth conditions: control (25 µM FeNaEDTA); −Fe (0.1 µM FeNaEDTA); −Fe (0.1 µM FeNaEDTA) and sodium nitroprusside (SNP, 100 µM) as nitric oxide (NO) donor; and SNP (100 µM). The pictures of the stained roots were taken at 10× magnification. Scale bar = 100 μm.

Endogenous NO level in alfalfa root was slightly induced in Fe deficient condition that was lower compared to SNP treated plants. However, the addition of SNP in combination with or without Fe deficient plants showed a strong green fluorescent signal compared to control. The non-treated control plant exhibited very weak fluorescence intensity in alfalfa root (Figure 5).

### 3.5. Regulation of Mineral Nutrition and Transporter Expression

**Figure 6:**
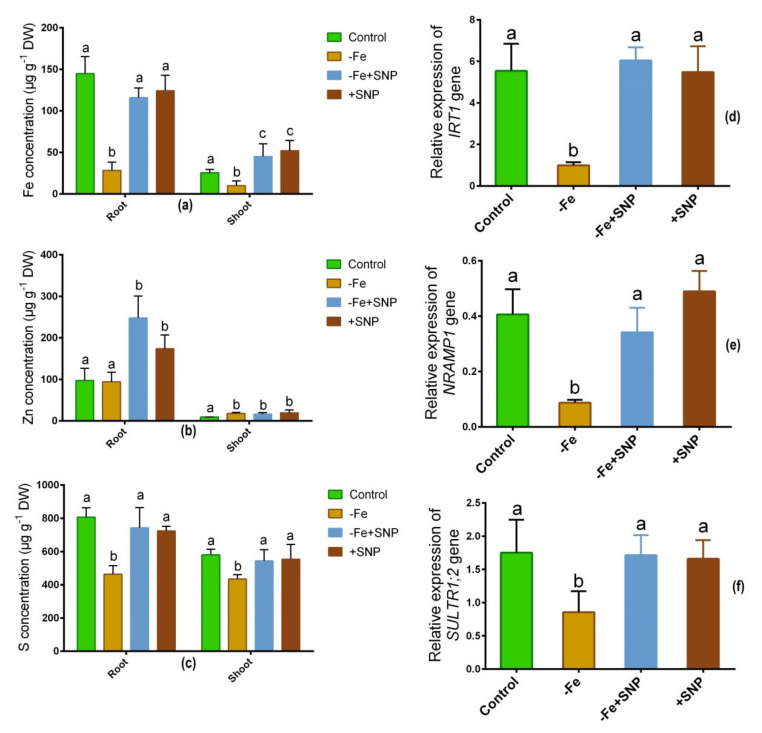
Fe (**a**), Zn (**b**) and S (**c**) concentration, along with *IRT1* (**d**), *NRAMP1* (**e**), and *SULT1;2* (**f**) candidate gene expression in alfalfa cultivated with different growth conditions: control (25 µM FeNaEDTA); −Fe (0.1 µM FeNaEDTA); −Fe (0.1 µM FeNaEDTA) and sodium nitroprusside (SNP, 100 µM) as nitric oxide (NO) donor; and SNP (100 µM). Different letters above the error bar indicate significant differences (*p* < 0.05) among means ± SD of treatments (*n* = 3).

Fe deficiency-regulated the concentrations of Fe, Zn, and S in alfalfa roots and shoots. Fe level was significantly decreased in Fe-deficient condition in root and shoot while it was increased after SNP supplementation (Figure 6a). Zn concentration was unchanged in roots under Fe-deficient and control condition but it was elevated in response to SNP-treated roots and shoots (Figure 6b). The S level was significantly reduced both in roots and shoots under Fe-deficiency (Figure 6c). However, neither plants treated with SNP nor untreated control plants had significantly different S concentrations in their roots or shoots.

As a consequence, several candidate genes involved in transporting metallic ions expressed differently in alfalfa roots and shoots. The Fe regulating gene *IRT1* exhibited subtaintial downregulation in response to Fe deficiency, and demonstrated significant upregulation in response to −Fe+SNP or +SNP treatments (Figure 6d). Fe-deficiency induced-stress significantly declined the expression of *NRAMP1* while the expression of the gene enhanced after SNP supplementation (Figure 6e). Sulfate transporter gene *SULT1;2* showed a significant downregulation in Fe-deficiency stress. However, the expression of *SULT1;2* was not significantly different among the −Fe+SNP, +SNP and non-treated control plants (Figure 6f).

### 3.6. Antioxidant Enzyme Activity

**Figure 7:**
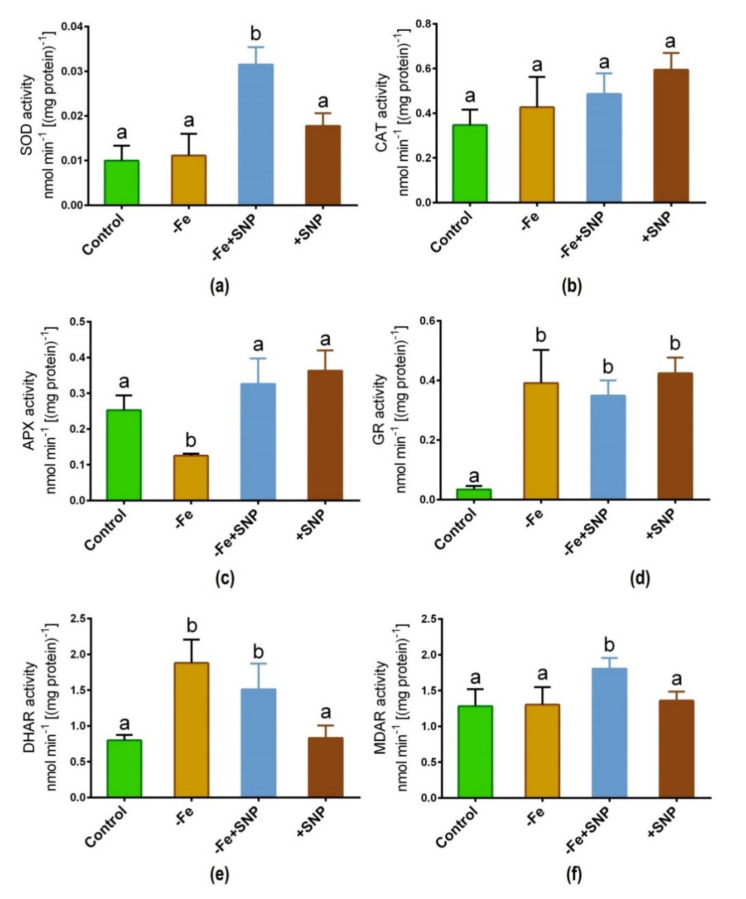
SOD (**a**), CAT (**b**), APX (**c**), GR (**d**), DHAR (**e**) and MDAR (**f**) enzyme activity in alfalfa cultivated with different growth conditions: control (25 µM FeNaEDTA); −Fe (0.1 µM FeNaEDTA); −Fe (0.1 µM FeNaEDTA) and sodium nitroprusside (SNP, 100 µM) as nitric oxide (NO) donor; and SNP (100 µM). Different letters above the error bar indicate significant differences (*p* < 0.05) among means ± SD of treatments (*n* = 3).

Fe deficiency-induced stress along with SNP supplementation significantly regulated the activity of key antioxidant enzymes in alfalfa. The addition of SNP to Fe deficient conditions led to a significant increase in SOD activity, although this increase was not consistent across the different treatments (Figure 7a). CAT activity was slightly lifted in SNP-treated plants, but this enzyme did not exhibit significant changes due to Fe deficiency or SNP treatments (Figure 7b). Fe deficiency showed a significant decrease in APX activity while it increased and it was consistent in SNP treated (+SNP) plants (Figure 7c). GR activity was greatly increased under Fe deficiency with or without SNP supplementation compared to non-treated control (Figure 7d). DHAR showed a significant increase in its activity in Fe-deficient condition, along with Fe deficiency with SNP (−Fe+SNP) treatment (Figure 7e). Furthermore, significant MDAR activity was observed only in Fe deficiency with SNP of all assayed treatments (Figure 7f).

### 3.7. Expression of Key Genes Involved in ROS Homeostasis

**Figure 8:**
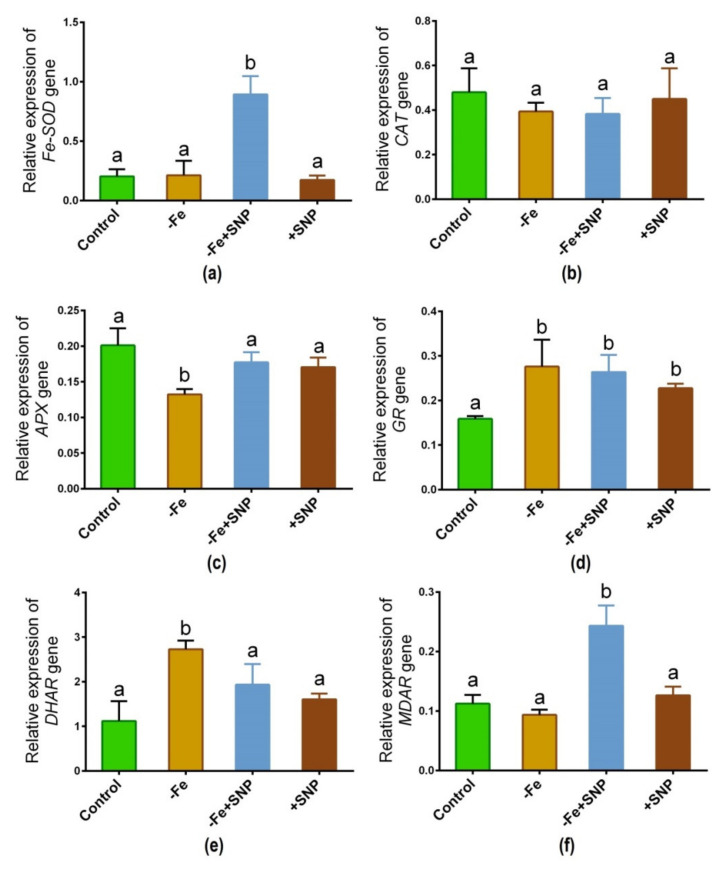
*Fe-SOD* (**a**), *CAT* (**b**), *APX* (**c**), *GR* (**d**), *DHAR* (**e**) and *MDAR* (**f**) candidate gene expression in alfalfa cultivated with different growth conditions: control (25 µM FeNaEDTA); −Fe (0.1 µM FeNaEDTA); −Fe (0.1 µM FeNaEDTA) and sodium nitroprusside (SNP, 100 µM) as nitric oxide (NO) donor; and SNP (100 µM). Different letters above the error bar indicate significant differences (*p* < 0.05) among means ± SD of treatments (*n* = 3).

The expression of major antioxidative enzyme genes is regulated differentially in response to Fe-deficiency with or without SNP supplementation. As shown in Figure 8, the transcript of the *Fe-SOD* gene significantly was increased due to the addition of SNP to the Fe deficient (−Fe+SNP) condition (Figure 8a). *CAT* gene showed the expression with nearly the same pattern among the treatments (Figure 8b). Fe-deficiency significantly declined the expression of the *APX* gene, while the pattern remained unchanged in the rest of the treatment groups (Figure 8c). Interestingly, *GR* gene showed a significant up-regulation under Fe-deficiency along with SNP-treated plants compared to control (Figure 8d). Ascorbate-glutathione (AsA-GSH) cycle gene *DHAR* is significantly upregulated in response to Fe deficiency (Figure 8e). However, the combination of SNP with Fe deficiency highly induced the transcript of *MDAR* gene, while the rest of the treatments showed nearly the same pattern (Figure 8f).

## 4. Discussion

This study showed a mechanistic basis related to NO-mediated protection of chlorosis, photosynthetic disturbance, and oxidative stress in alfalfa. The role of NO and its signaling response in alfalfa roots reveals novel features along with antioxidant enzymes and corresponding candidate genes linked to the protection of plants from Fe deficiency- induced damages. These findings can be utilized by the farmer to improve alfalfa production in Fe-deficient soils.

### 4.1. NO Mitigated Chlorosis, Photosynthetic Disruption, and Plant Growth Reduction

In Fe-deficient condition, a reduction of root-shoot biomass, chlorosis, and photosynthetic disturbance was observed, which confirming the inhibition of morph physiological features is the adverse effects of Fe-deficiency in plants. Reduction of chlorophyll synthesis in chlorosis leaves, fresh weight, and photosynthetic rate is associated with Fe-deficiency [23]. However, chlorosis is also involved in reducing Fe accumulation and other nutritional imbalances that are related to growth retardation in alfalfa. Another report suggests that nutrient deficiencies caused by Fe-deficiency have an impact onplant growth and development [24]. Our study indicates that NO is capable of coping with Fe-deficiency as well as growth retardation, which demonstrates the role of NO in Fe homeostasis particularly in Fe deficient plants [25]. NO also protects the photosynthetic disturbance by regulating the leaf greenness in alfalfa. However, it is well documented that NO protects against photosynthetic disturbance in plants under stressful conditions [26]. In this study, the ratio of Fv/Fm manifested a remarkable reduction due to Fe deficiency. It indicates that the PSII photochemical reaction considerably affected growth attributes, which impaired the initial growth phase of Fe-deficiency in alfalfa. This is supported by the evidence in plants where reduction of PSII is often associated with Fe-deficiency in leaves [27]. A study documented that the redox state of PSII acceptors was negatively influenced by Fe-deficiency [28]. In contrast, the quantum yield of PSII was well maintained following supplementation of NO in alfalfa. It implies that Fe-deficiency inhibits the Fe uptake but Fe acquisition can be well maintained by NO. Thus, it suggests that NO may play an important role in maintaining photosynthesis capacity and it is required for the mechanism involved in Fe-deficiency tolerance in alfalfa.

### 4.2. Endogenous NO Level Reduced ROS-Induced Cellular Damages

In plants, Fe deficiency causes reactive oxygen species (ROS), cellular injury, and non-autolytic programmed cell death (PCD) [29]. In this present study, Fe-deficiency induced ROS (H_2_O_2_) level, increased cell death percentage (%). At the same time, these parameters significantly recovered after addition of SNP. Thus, it suggests that Fe-deficiency stress linked to ROS generation and oxidative stress, which involved in cellular injury in alfalfa. In contrast, alleviation of Fe deficiency and reduction of ROS induced cellular damage by NO suggests that generation of endogenous NO (detected using DAF-2 DA fluorescence probe) in root cells is associated with Fe-homeostasis. A recent study evidenced that NO is involved in Fe-homeostasis especially in Fe deficient plants [25]. Hence, low intensity of temporal accumulation of NO is also visualized in Fe-deficient alfalfa roots.It is not surprising that NO signal can be induced in plant due to Fe-deficiency [29].

### 4.3. NO Involved in Regulating Nutrients Accumulation and Transporter Gene Expression

The retardation of alfalfa growth with chlorotic symptoms as a consequence of low Fe accumulation may be associated with lower uptake and translocation of Fe in Fe- deficient plants. However, this occurrence did not happen in the case of SNP treated plants. It is evidenced that *IRT1* gene is involved in transporting Fe in the cytosol of cells [30]. In Arabidopsis, it is also documented that *IRT1* transporter expressed in the root, and it is responsible for Fe uptake from the soil for plant growth [31]. In this study, *NRAMP1* gene significantly downregulated in Fe-deficiency but sharply induced after the addition of SNP with Fe deficiency, indicating that *NRAMP1* is associated with the low Fe uptake and transport. In contrast, the induction of *NRAMP1* due to SNP supplementation suggests that it may be involved in Fe homeostasis, as it is evidenced that *NRAMP1* is a crucial metal transporter involved in Fe transport as well as homeostasis in plants [32]. In addition, *SULTR1;2* gene associated with sulfate transport showed increased expression due to SNP supplementation and declined to Fe deficiency. However, it has yet to disclose *SULTR* gene function in *Medicago* species, *SULTR* study in legume plant *Lotus japonicas* suggested that *LjSULTR1:2* is involved in sulfate uptake [33].Consequently, knockout of *SULTR1;2* was reported to decline sulfate uptake and growth in Arabidopsis [34]. This evidence suggests that S accumulation is linked to the response of *SULTR1;2* and activity depend on Fe availability in plants.

### 4.4. Antioxidant Genes Expressions Was Tightly Related to the Changes of Corresponding Enzymes Activities

An intimate relationship between antioxidant enzyme activities and Fe deficiency-induced stress was reported in soybean plants [35]. In this study, we found that ROS scavenging-related genes expressions were tightly related to the alterations of corresponding enzymes activities in alfalfa. Changes in leaf greenness and gene expression indicate the disturbance of chloroplast integrity under Fe deficiency. Fe can interact with *SOD* as a co-factor, so it is expected that a possible interaction exists between the response of *Fe-SOD* and Fe availability/efficiency in plants. Fe-SOD enzyme involved in detoxification of superoxide formed during photosynthetic electron transport and function in ROS metabolism [36]. Fe-availability is a vital determinant of Fe-SOD expression. As analysis of alfalfa phenotype provides support for the role of this enzyme in ROS scavenging. In this study, Fe-deficiency declined the expression of *Fe-SOD* transcript but was significantly induced after supplementation of SNP in alfalfa. Our findings supported the study in the *Arabidopsis* plant where *Fe-SOD* transcript was downregulated in response to Fe deficiency [37]. *CAT* expression and its corresponding enzyme activity were not significantly increased, though slightly induced in response to SNP, indicating that CAT may not be actively involved in particularly Fe-deficiency induced stress alleviation due to overproduction of ROS in plants [38].

**Figure 9:**
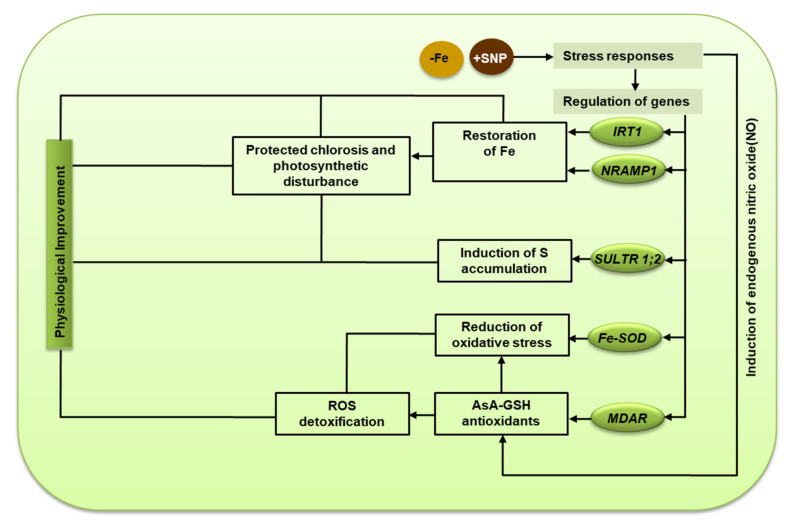
The mechanistic basis of nitric oxide-mediated protection of Fe deficiency-induced chlorosis, photosynthetic disturbance, and oxidative stress in alfalfa.

The activity of the Fe-containing enzyme APX was lower in Fe-deficiency stress but it tended to be induced by SNP. This finding indicates that a low level of Fe in tissue possibly influenced the activity of the APX enzyme along with the expression of APX transcript. However, the high *Fe-SOD* and *APX* activity presence of SNP indicates that *Fe-SOD* and *APX* improve alfalfa to reduce ROS-induced oxidative injury in plants. The report suggests that Fe deficiency leads to chlorosis, ROS generation, and oxidative stress, which induce cellular injury and non-autolytic PCD in plants [6]. In this study, the supplementation of SNP stimulated the transcripts of *GR*, *DHAR*, and *MDAR* as well as their corresponding enzyme activities, part of the ASC-GSH pathway. These comprehensive insights suggest a mechanistic basis of SNP-mediated protection of Fe deficiency-induced chlorosis, photosynthetic disturbance, and oxidative stress in alfalfa (Figure 9).

## 5. Conclusions

The results of this study shed light on the mechanisms that underlie SNP-mediated alleviation of Fe deficiency-induced growth retardation, chlorosis, photosynthetic disturbance, and oxidative stress in plants. Fe deficiency-induced chlorosis is one of the consequences of Fe deficiency when plants are grown in such a condition. In this study, Fe-deficiency induced stress significantly impacted root-shoot length, leaf greenness, maximum quantum yield PSII (Fv/Fm), Fe, S, and Zn accumulation, increased H_2_O_2_ content in alfalfa. Surprisingly, these negative impacts of Fe-deficiency stress were largely restored due to SNP supplementation. The response of Fe and S transports in under Fe-deficiency suggested that Fe shortage declined the Fe, Zn and S accumulation both in root and shoots as well as significant decreased of *IRT1*, *NRAMP1* and *SULTR 1;2* gene transcripts in alfalfa. Furthermore, SNP-induced antioxidant candidate genes along with their corresponding enzyme activities indicate that SNP-induced antioxidant enzymes are involved in preventing of Fe deficiency stress-induced chlorosis, photosynthetic disturbance, and ROS accumulation as well as oxidative stress in alfalfa.

## Data Availability

Data are contained within the article and Supplementary Materials.

## Supplementary Materials

The following are available online at www.mdpi.com/article/10.3390/antiox10101556/s1, Table S1: Primer sequences used in qRT-PCR.
