# Supplementary material for: Nitric Oxide Prevents Fe Deficiency-Induced Photosynthetic Disturbance, and Oxidative Stress in Alfalfa by Regulating Fe Acquisition and Antioxidant Defense
Source: Antioxidants (Basel). 2021 Sep 29;10(10):1556. doi: 10.3390/antiox10101556 (PMC8533379; doi:10.3390/antiox10101556)
Supplement: Supplementary file 1 [file antioxidants-10-01556-s001.zip › antioxidants-1381694-supplementary.pdf]

Supplementary Table S1. Primers sequences used for qPCR analysis

Gene name	Accession no	Primers	Sequences
MsActin	JQ028730.1	Forward	TTCTCACCACACTTCTCGCC
		Reverse	CCAGCCTTCACCATTCCAGT
MsFeSOD	AF377344.1	Forward	GAGTACCATTGGGGAAAGCA
		Reverse	CCATACCTGTGCTGCATTGT
MsAPX	EX522382.1	Forward	GAAATGCGCTCCTCTTATGC
		Reverse	TGTTAGCACCATGAGCAAGC
MsMDAR	EX522162.1	Forward	TGGTCTACCCAGAACCTTGG
		Reverse	CCAGTACCCTTCCGTCCTTT
MsGR	AM407889.2	Forward	AGGACGGTGAACCTGATTTG
		Reverse	TCTAGCAGCACGAACACCAC
MtIRT1	KX641478.1	Forward	TTTACCCTTGGCGACACGTT
		Reverse	CATGAACCCGGTCCCAAGAA
MtNramp1	XM_024781241.1	Forward	GCATTGCTAGCCTCAGGACA
		Reverse	TCCATGCGTATGCAGGTGAT
MtSULTR1;2	*Medtr3g073780*	Forward	TATTATCTCCGTGTTGAAGGC
		Reverse	CAATAAATTTGGCGACCAG
MtCAT	*XM_013606824.1*	Forward	CCAAGTCCCACATTCAGGAG
		Reverse	ACTGCTTTCCCAGCCTTGTT
MtDHAR	*DQ006811.1*	Forward	GTGTTGCTGACACTGGAGGA
		Reverse	CCAGCTGTAGCCTTTTCAGG
